# Impact of Hydrotherapy on Antioxidant Enzyme Activity in an Elderly Population

**DOI:** 10.3390/geriatrics7030064

**Published:** 2022-06-13

**Authors:** Ana Valado, Stephanie Fortes, Márcia Morais, Rogério Barreira, João Paulo Figueiredo, Armando Caseiro

**Affiliations:** 1 Biomedical Laboratory Sciences, Coimbra Health School, Polytechnic Institute of Coimbra, Rua 5 de Outubro—SM Bispo, Apartado 7006, 3045-043 Coimbra, Portugal; fannylima97@gmail.com (S.F.); marciad.morais1@gmail.com (M.M.); rogerio.barreira@estesc.ipc.pt (R.B.); armandocaseiro@estesc.ipc.pt (A.C.); 2 Laboratory for Applied Health Research (LabinSaúde), Rua 5 de Outubro-SM Bispo, Apartado 7006, 3046-854 Coimbra, Portugal; 3 Marine and Environmental Sciences Centre (MARE), Faculty of Sciences and Technology, University of Coimbra, 3001-456 Coimbra, Portugal; 4 Centro Hospitalar e Universitário de Coimbra, Serviço de Sangue e Medicina Transfusional, Praceta Prof. Mota Pinto, 3000-075 Coimbra, Portugal; 5 Complementary Sciences, Coimbra Health School, Polytechnic Institute of Coimbra, Rua 5 de Outubro—SM Bispo, Apartado 7006, 3045-043 Coimbra, Portugal; jpfigueiredo@estesc.ipc.pt; 6 Unidade I&D Química-Física Molecular, Faculdade de Ciências e Tecnologia, Universidade de Coimbra, 3030-790 Coimbra, Portugal

**Keywords:** hydrotherapy, aging, oxidative stress, superoxide dismutase, glutathione peroxidase, glutathione reductase

## Abstract

Oxidative stress is defined as the imbalance between reactive species and antioxidant agents. One of the effects of oxidative stress is the normal process of cellular aging that stems from the accumulation of tissue damage. Epidemiological studies show that regular physical exercise prevents the injuries caused by aging. The objective was to evaluate whether the practice of hydrotherapy, in an elderly population, positively influenced the activity of the enzymes superoxide dismutase, glutathione peroxidase and reductase that act by reducing reactive species in the body. The study involved 37 participants aged ≥ 60 years, of both sexes, divided into experimental and control groups. The experimental group performed 15 hydrotherapy sessions. Enzyme activity was evaluated in two moments: T0-before the first session, and T1-after the last session, with blood collections conducted in both. In T1, there was a significant increase vs. T0 of glutathione peroxidase activity (57.72 ± 19.99 vs. 48.14 ± 17.22 U/g Hb) and glutathione reductase activity (100.18 ± 30.85 vs. 78.44 ± 21.26 U/L). Both sexes tended to show higher values at T1. We concluded that hydrotherapy proved to be a positive stimulus for the enzymatic antioxidant activity of the elderly, suggesting that a regular and moderate practice of physical exercise induces better and higher quality of life.

## 1. Introduction

Oxidative stress is defined as the imbalance between reactive species produced in the body during metabolic reactions and the antioxidant agents that could neutralize or prevent the formation of these species. The antioxidant system consists of enzymatic and non-enzymatic systems [1]. The enzymatic antioxidant system is composed of the following enzymes: superoxide dismutase (SOD), catalase (CAT), glutathione peroxidase (GPx), and glutathione reductase (GR) and it is the main system against reactive oxygen species (ROS). The non-enzymatic antioxidant system, in addition to providing direct protection to the body against oxidative damage, can also increase the function of the enzymatic system. This system is made up of molecules such as vitamins (C and E), phenolic compounds, flavonoids, and carotenoids found naturally in foods [2]. Oxidative stress acts out directly on the normal biomolecules (lipids, proteins and DNA) that can trigger pathologies and affect the normal process of cellular aging [1]. Recent studies confirm the direct relationship between oxidative stress consequences and chronic diseases, specifically neurodegenerative, cardiovascular, and inflammatory diseases, diabetes, and cancer [1,3,4]. ROS are generated from molecular oxygen as a result of cellular processes and can be divided into the following two groups: free radicals (contain one or more unpaired electrons in the last electronic layer) and non-radical forms (two free radicals share their unpaired electrons). The main endogenous oxidants are superoxide anion (O_2_^−^∙), hydrogen peroxide (H_2_O_2_), and hydroxyl radical (OH∙). Smoking habits, ozone exposure, hyperoxia, exposure to ionizing radiation, and some heavy metal ions can also contribute to the formation and/or increase of ROS [3].

The metal ion-dependent superoxide dismutase enzymes are a group of enzymes that are responsible for the dismutation of two superoxide anions into hydrogen peroxide and oxygen. There are three members of the SOD family, namely two copper and zinc-dependent superoxide dismutase, one present mainly in the cytoplasm (SOD1) and the other extracellular (SOD3) and there is also manganese-dependent superoxide dismutase (SOD2), present in the mitochondrial matrix [5]. Deficiencies in these isoforms have been associated with the development of arterial hypertension, diabetes, and heart failure [6]. The practice of physical exercise induces adaptive antioxidant responses mediated by mitogen-activated proteins (MAPK p38, ERK 1 and ERK 2) and by redox-sensitive transcription factors (NF-kB), which increase SOD expression and activity [7].

Glutathione (GSH) is a tripeptide present in all human cells formed by the following three amino acids: glycine, glutamate, and cysteine. Its synthesis occurs in the cytoplasm, where it is more distributed; however, it can be found in smaller amounts in other organelles. It is one of the main antioxidants present in the cytoplasm and its functions are to directly remove free radicals and/or indirectly support the enzymatic activity as a cofactor. Intracellular GSH, in its reduced form, is a monomer and after its oxidation gives rise to the GSSG dimer (oxidized form) [8,9]. This oxidation occurs in the presence of ROS in which they donate an electron to GSH [10]. The enzyme GR has the function to revert GSSG into GSH. It is considered the main redox buffer of the cell and the ratio GSH/GSSG is considered as a biomarker of oxidative stress [8]. GPx is a selenium-dependent enzyme whose function is to reduce hydrogen and lipid peroxides. When it is present in reduced amounts, and there is a presence of high amounts of H_2_O_2_, an imbalance occurs in the body that can cause tissue damage and the activation of inflammatory pathways related to nuclear factor-κB (NF-κB) [11]. This enzyme is involved in H_2_O_2_ homeostasis and uses GSH as a substrate [12,13].

Aging can be defined as a complex biological process [2] in which there is a progressive loss in physiological functions after the reproductive phase of life [14]. There are several theories that attempt to explain the aging process and the free radical theory is the most accepted. This theory is based on the fact that the production of free radicals during aerobic metabolism can contribute to the development of DNA, lipid, and protein damage [14]. The overload of the antioxidant defense mechanisms, as a consequence of high levels of ROS, also contributes to the functional loss of cells [7]. Epidemiological data reveal that the regular practice of physical activity contributes to preventing the dangerous effects caused by aging, playing antioxidant roles [7].

The practice of physical activity, specifically water-based exercises, shows a lower risk of bone fractures and articulations are less exposed to stress and to impact compared to land-based exercises. These exercises are highly recommended for older people, especially those with limited movement [15], because they present a low load and mechanical stress on weight-bearing articulations and muscles [16]. Hydrotherapy consists of a set of exercises with physiotherapist purposes, essential in the rehabilitation of functional changes in the body. In addition, there are the physical, physiological, and kinesiological effects obtained by immersing the body in a swimming pool with heated water. Considering the physical properties and water temperature, these exercises contribute to the improvement and maintenance of the range of articulations movement, relaxation and reduction in muscle tension [17], improvement in cardiovascular functions and also in reducing pain [16]. According to Da Silva et al., in 2017, it was concluded that low-intensity aquatic exercise programs improve levels of oxidative stress, anxiety, functional autonomy, and inflammation in hypertensive patients and suggest that aquatic physical exercise should be considered as an common physical exercise intervention for the control of arterial hypertension and the improvement of the quality of life [18]. Water-based exercises have been shown to significantly increase the effectiveness of physical training in patients with chronic obstructive pulmonary disease [19] and a hydrotherapy session can reduce arterial stiffness in pregnant women with high-risk chronic arterial hypertension [20]. The practice of hydrotherapy has also been shown to contribute to symptom relief in patients with musculoskeletal disorders, functional improvement in patients with neurological disorders, and the rehabilitation of patients with acute injuries. As it has a positive effect on disease prevention, treatment, and rehabilitation, it is expected that this type of exercise will be applied as an effective health promotion program [21] for the benefit of the general population. In addition to providing health benefits, the practice of physical activity can increase the levels of oxidative stress biomarkers available in the body, and currently, there are no studies that explain these two paradoxical findings [22].

The present study aimed to evaluate whether the practice of hydrotherapy, in an elderly population, positively influenced the activity of SOD, GPx and GR enzymes.

## 2. Materials and Methods

### 2.1. Study Design

The study comprised 37 participants aged between 60 and 89 years of both sexes who were divided into the following two groups: experimental and control. The groups were made up of patients from a rehabilitation center in Portugal. Experimental group involved patients undergoing treatment, with a medical prescription for the practice of hydrotherapy, while the control group did not perform hydrotherapy exercises. All participants had comorbidities and were medicated. Participation in the study had as an inclusion criterion a minimum age of 60 years, and the volunteers in the experimental group had no impediment in the practice of physical activity. In the design of the study it was presented (n = 50), however only 37 participants completed the investigation.

### 2.2. Hydrotherapy

The hydrotherapy involved a total of 15 classes with aquatic exercises in a therapeutic pool. There were 2 classes per week lasting 30 min each. The classes had a sequence of varied movements distributed over 3 moments: warm-up, aerobic exercise, relaxation and always in the presence of a physiotherapist. Therapeutic exercises follow the guidelines of the Aquatic Exercises Association, which recommends an aquatic exercise protocol for specific populations, implemented in a pool with a temperature adjusted to 33 ± 1 °C and the depth of the water was set at the xiphoid process level, well as the outside environment [16]. The study consisted of two distinct moments: T0, before the intervention and T1, after the last hydrotherapy session.

### 2.3. Sample Collection and Processing

Blood samples were collected from the veins of the participants’ median cubital fossa. In the experimental group, two samples were collected (K_3_ EDTA tube and gel tube) at each moment (T0 and T1). All samples were centrifuged at 1800× *g* for 10 min at 4 °C and then aliquoted into plasma and serum. Then, the aliquots were stored at −20 °C until the biochemical assays were performed. For the control group, a similar procedure was performed in T0 moment.

SOD, GPx and GR enzyme activities were determined using the commercial RANSOD Superoxide Dismutase, RANSEL Glutathione Peroxidase and Glutathione Reductase kits from Randox Laboratories Limited, UK. All samples were performed in duplicate and the procedure described in the kit was followed. To quantify the hemoglobin concentration, to normalize the SOD and GPx values, the Cell-Dyn Sapphire™ hematology analyzer, Abbott Laboratories, Santa Clara, California, United States of America was used. SOD and GPx enzymatic activities were determined in red blood cells, while GR activity was determined in serum. To obtain the results, spectrophotometric readings were used at 505 nm for SOD and at 340 nm for GPX and GR (Multiskan™ GO Microplate Spectrophotometer, Thermo Scientific™, Vantaa, Finland). The SOD and GPx results were expressed in U/g Hb, while the GR was presented in U/L.

### 2.4. Statistical Analysis

All analyses were performed using IBM SPSS Statistics, version 25.0. A descriptive analysis was applied to characterize the sample through sociodemographic data and then an inferential analysis through statistical tests: *t*-Student for two paired samples, *t*-Student for two independent samples, *t*-Wilcoxon, correlation of Pearson and Wilcoxon–Mann–Whitney. Data were expressed as means ± standard deviation and the level of significance established for the test was *p* < 0.05, with a confidence level of 95%.

### 2.5. Ethical Considerations

This study was carried out according to the principles of the Declaration of Helsinki and approved by the Ethics Committee of Polytechnic Institute of Coimbra (45/2018). The participation of all patients was voluntary, with previous knowledge of this study. All participants provided their sociodemographic information and written consent before participation.

## 3. Results

### 3.1. Characterization of the Patients

According to sociodemographic data, the experimental group was 68.37 ± 5.21 years old and 88.9% were females (Table 1). A total of 75% of these patients had chronic pathologies such as hypertension, rheumatoid arthritis, arthrosis, diabetes mellitus, muscle and bone pain, cardiomyopathies. In addition, 40% of participants reported taking more than 6 drugs a day and 57.2% rated their health status as “reasonable”.

The control group was 74.5 ± 6.69 years and 80% were female (Table 1). All patients had the various pathologies mentioned above. A total of 50% of the participants classified their health status as “reasonable” and regarding their consumption of drugs, 44.4% stated that they took between 4 and 6 drugs a day.

### 3.2. Superoxide Dismutase Activity

A comparative analysis of SOD enzymatic activity was performed between the control and experimental groups, at T0 and T1. The experimental group recorded 1437.64 ± 593.46 U/g Hb at T0 and 1421.41 ± 705.39 U/g Hb after the hydrotherapy intervention, without statistically significant differences. Pearson’s correlation test was applied to measure the degree of association between age and enzyme activity and there was no correlation between these two variables (*p* = 0.292). Then, the Student t test was used to compare the means of enzymatic activity with sex, verifying that men tended to have lower values in T1 (1298.34 ± 673.60 vs. 1546.84 ± 188.60), without statistically significant differences (*p* > 0.05).

### 3.3. Glutathione Peroxidase Activity

**Figure 1:**
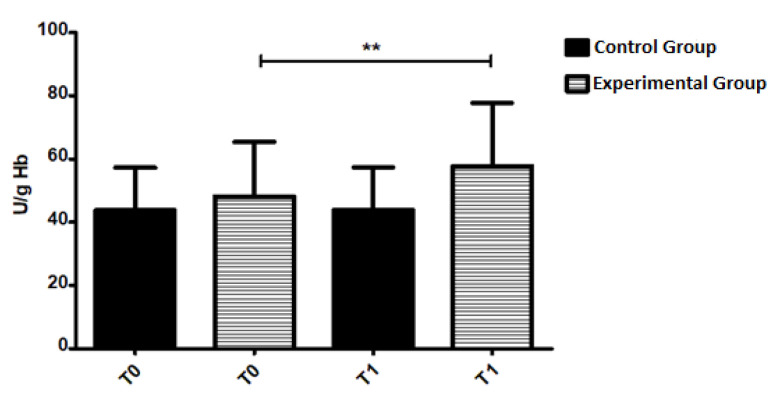
Glutathione Peroxidase activity at T0 and T1 of control and experimental groups. The results are expressed as U/g Hb and as mean ± standard deviation. ** *p* < 0.005.

In the analysis of the results of GPx activity between the control and experimental groups, there was a trend towards increased activity in the experimental group at T1 (*p* = 0.05) (Figure 1). It was observed that the GPx activity in experimental group increased from 48.14 ± 17.22 U/g Hb to 57.72 ± 19.99 U/g Hb after the hydrotherapy intervention (*p* = 0.0015, Figure 1). Using the t-Wilcoxon test, we confirmed that 19 patients showed this statistically significant increase at T1. Pearson’s correlation was tested to correlate enzymatic activity with the age of the patients and a positive correlation was observed, but not reaching statistical significance (*p* = 0.261).

### 3.4. Glutathione Reductase Activity

**Figure 2:**
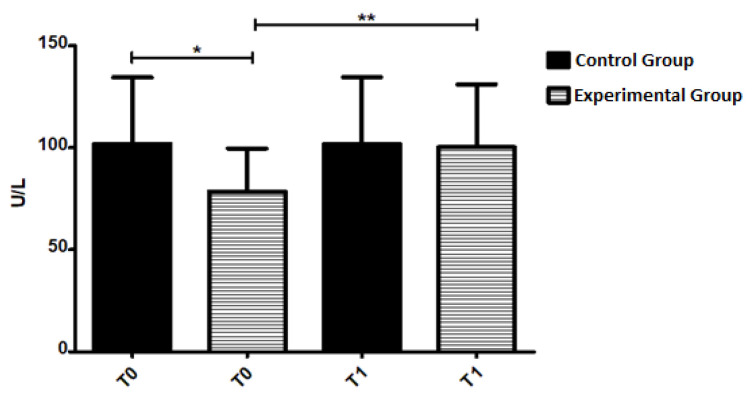
Glutathione Reductase activity at T0 and T1 of control and experimental groups. The results are expressed as U/L and as mean ± standard deviation. * *p* < 0.05, ** *p* < 0.005.

The comparative analysis of the GR results between the control and experimental groups suggests differences at T0. The GR activity results in the experimental group showed a significant increase in T1 (*p* = 0.0005, Figure 2) compared to T0 (100.18 ± 30.85 U/L vs. 78.44 ± 21.26 U/L). Pearson’s correlation test was applied to compare the enzyme activity with the age of the patients and showed a negative correlation, but without statistical significance (*p* = 0.650).

## 4. Discussion

One of the consequences of oxidative stress is the normal and physiological process of cellular aging that arises from the accumulation of tissue damage caused by free radicals [2]. In addition to certain diets [2], the practice of physical exercise can also increase antioxidant functions and protect the body against oxidative damage, through SOD enzymes (first route of defense against free radicals), CAT, GPx and GR [23], thus preventing premature aging. Some studies suggest that the regular practice of physical exercise [24], such as aquatic exercises [18,25], positively restores the vascular capacity in older and sedentary people and also improves anxiety, functional autonomy, and reduces oxidative stress and inflammation in hypertensive patients [25]. Da Silva and co-authors suggest that aquatic physical exercises should be considered as a common physical intervention for the control of arterial hypertension and other cardiovascular diseases and the improvement of the quality of life [18,25]. The practice of moderate aerobic physical exercises can upregulate the various antioxidant genes, and therefore play antioxidant functions [26]. The MnSOD enzyme contains NF-κB binding sites in its gene promoter region, so it may be a target for exercise-activated upregulation through the NF-κB signaling pathway [27]. The levels of antioxidants increase as they act as an adaptation mechanism mediated by the redox balance, as a way of protecting the body against damage caused by the high amounts of oxygen produced after physical exercise [23]. Therefore, it is believed that the practice of physical exercise is two sided, that is, it brings health benefits but can be harmful if oxidative stress levels exceed healthy limits [22]. The purpose of the study was to estimate the effect of hydrotherapy exercises on oxidative stress, through the enzymatic activity of SOD, GPx and GR. Aquatic exercises were chosen because they have a lower risk of bone fractures, low load and mechanical stress on weight-bearing joints and muscles when compared to land-based exercises and because they are strongly recommended for the elderly, especially those with movement limitations [15,16]. Regarding SOD enzymatic activity, our results did not present statistically significant differences at T1. Possibly, the exercise plan performed (number of sessions, frequency of sessions per week, and exercise intensity) was not sufficient to enhance the SOD activity. Additionally, the presence of sufficient substrate could be a crucial factor for the increase of enzyme activity. Bouzid et al., in 2013, compared the levels of different biomarkers of oxidative stress between a young group and a group of elderly people, at rest and after a gradually increasing exhaustive exercise. They revealed that SOD activity, after exercise, only increased in the young group and suggested that aging may be responsible for the decrease in the protein content of enzymes, and consequently affect the enzymatic activity [28]. In 2016, Okudan and Belviranli carried out a study where they evaluated the effects of physical exercise on hepatic oxidative stress and antioxidant status in aged mice. They found that the generation of free radicals increases with age and, consequently, there is a decrease in SOD activity. They suggested that this event depends on the degradation of the enzyme induced by ROS or the decrease in its synthesis due to the aging process [29]. Moreover, by correlating sex with the activity of this enzyme, we observed that female subjects tended to have higher levels of enzyme activity in T1. In 2013, Rowinski et al. evaluated the relationship between markers of oxidative stress and the activity of antioxidant enzymes and physical activity in elderly males and females and found that female subjects had higher SOD values than males. These same investigators also observed that the worsening of antioxidant status was directly related to advancing age [30]. Yu and co-authors performed a study in 2018 that investigated the effect of different types and frequencies of physical exercises on blood pressure, also evaluating the levels of oxidative stress biomarkers in elderly hypertensive subjects. In contrast to other studies, they observed that subjects who performed exercises demonstrated decreased levels of malondialdehyde (a specific biomarker of lipid peroxidation) and increased SOD activity [24]. Gomes and collaborators, in 2016, evaluated the enzymatic activity of SOD in an elderly population subjected to a combined plan of several exercises for 12 weeks, followed by a break. They observed that the activity of this enzyme increased significantly after the intervention of physical exercises, concluding that this intervention stimulated the antioxidant capacity of the organism [31]. Bouzid and co-authors, in 2014, investigated the biomarkers of oxidative stress induced by rest and exercise in a sample of healthy elderly subjects undergoing low-intensity aerobic exercises. They demonstrated that the active group presented better results of SOD activity at rest and during recovery compared to the sedentary group and raised the hypothesis that active elderly subjects have a greater ability to prevent the production of superoxide anion [32]. According to our results, we observed that the activity of the GPx and GR enzymes increased significantly after the practice of hydrotherapy in the experimental group. These findings are corroborated by Rowinski and co-authors who showed that the activity of these two enzymes increased in the physically active elderly group (male and female) [30]. Another investigation based on the practice of physical activity on a cycle ergometer, with groups of participants (sedentary young people, active young people, sedentary elderly people, active elderly people), before and after exercise, revealed an increase in GPx in active elderly people, after exercise [33]. New evidence with hydrotherapy practice in patients with rheumatoid arthritis (RA) used as a methodology associated with treatment with conventional RA drugs, for a period of 12 weeks, showed an increase in GPx activity [26], supporting our results. Sousa et al. evaluated the activity of GPx and GR in the elderly submitted to a physical exercise plan for 12 weeks followed by an interval. Additionally, contrary to our findings, they observed that both enzymes showed lower activity at T1 in relation to T0 and deduced that the volume and intensity of the exercises were not sufficiently robust to induce an increase in their activity [34]. They also showed that other factors, such as older age, the number of medications consumed daily, and the development of one antioxidant pathway more favorable over another, can lead to decreased glutathione enzymatic activity [34]. Regarding gender and enzymatic activity, both sexes tended to present higher values of these enzymes in T1 and, on the other hand, Rowinski showed that active elderly females showed better results for GR activity [30].

Once again, with this study, it was possible to show that the practice of exercise, especially hydrotherapy exercises, is favorable in the adaptation of antioxidant mechanisms.

The authors recognize some limitations to the study, namely the differences in the constitution of the experimental and control groups with respect to the presence of comorbidities associated with age and medications.

## 5. Conclusions

The study seems to confirm that the practice of hydrotherapy works as a stimulus for the enzymatic antioxidant system with increased activity of GPx and GR, which is very important in the aging process, reducing oxidant species. However, the researchers propose carrying out further studies, while taking into account the limitations of the study, to consolidate the findings obtained. Hydrotherapy should be considered a potential intervention for improving endogenous antioxidant defenses for the elderly.

## Figures and Tables

**Table 1:** Patient’s sociodemographic characterization.

		Experimental Group n = 27	Control Group n = 10
Age		68.37 ± 5.21	74.50 ± 6.69
Min/Max		60/82	67/89
Sex	Female	n = 24	n = 8
	Male	n = 3	n = 2

Min—minimum; Max—maximum; Results expressed as mean ± standard deviation.

## Data Availability

Not applicable.
